# Systemic Embolization and Myocardial Infarction due to Clinically Unrecognized Left Atrial Myxoma

**DOI:** 10.1155/2011/159024

**Published:** 2011-11-30

**Authors:** Britta Vogel, Dierk Thomas, Derliz Mereles, Wolfgang Rottbauer, Hugo A. Katus

**Affiliations:** ^1^Department of Cardiology, Medical University Hospital Heidelberg, Im Neuenheimer Feld 410, 69120 Heidelberg, Germany; ^2^Clinic of Internal Medicine II, University of Ulm, Albert-Einstein-Allee 23, 89081 Ulm, Germany

## Abstract

Myxomas are the most common primary tumors of the heart. We report an extraordinary severe case of left atrial myxoma, presenting with stroke, myocardial infarction, and multiple arterial embolism including aorta, splenic and renal arteries, and several peripheral arteries. The patient had previously been diagnosed with systemic vasculitis, a typical but less common finding caused by multiple emboli mimicking vasculitis. The myxoma was removed and atrial septum reconstruction was performed. In summary, early diagnostic differentiation of myxoma from vasculitis is critical, and immediate surgical removal of myxoma is required as the probability of thromboembolic complications increases over time.

## 1. Introduction

Myxomas are the most common primary tumors of the heart, usually occurring sporadically [1]. Rare hereditary cases of cardiac myxoma have been described as part of Carney complex [2]. Myxomas are often located in the left atrium where they arise mostly from the interatrial septum. Patients show varying clinical manifestations depending on size, location, and fragility of the tumor. Arterial embolism is a common complication of left atrial myxomas (40–65%), caused either by thrombus formation at the surface of the tumor or by fragmentation of villous myxomas with particularly fragile extensions [1, 3]. Cases of myocardial infarction, complete obstruction of the abdominal aorta, and aortic saddle embolism have been reported in association with cardiac myxoma [1, 4].

## 2. Case Presentation

**Figure 1:**
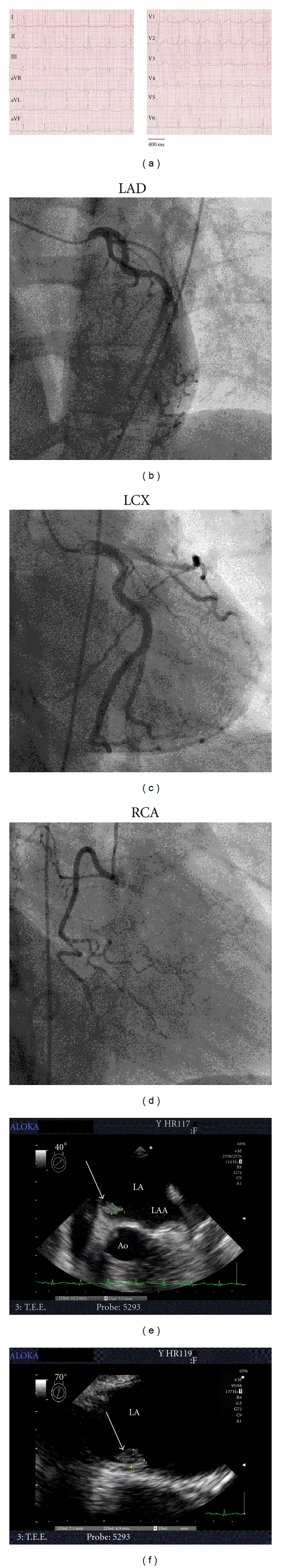
Findings obtained upon presentation with non-ST-elevation myocardial infarction. (a) 12-lead ECG on admission. (b–d) Coronary angiograms of left anterior descending artery (LAD; 25° left anterior oblique, 10° caudal angulation), left circumflex artery (LCX; 10° right anterior oblique, 30° caudal angulation), and right coronary artery (RCA; 30° right anterior oblique), revealing no signs of remaining thromboembolus or atherosclerosis. (e, f) Transesophageal echocardiograms, showing a left atrial tumor (arrow) located at the interatrial septum (40° (e) and 70° (f) angulation relative to the transversal plane, resp.). LA: left atrium; LAA: left atrial appendage; Ao: aorta. See text for details.

A 43-year-old female was transferred to our hospital with asymptomatic non-ST-elevation myocardial infarction (Figure 1(a)). Cardiac troponin T was elevated (0.32 *μ*g/L), and normal coronary angiography suggested embolic infarction (Figures 1(b)–1(d)). 

 Six weeks earlier the patient had been admitted to a stroke unit at an external hospital with hemiparesis of the right upper extremity, bilateral paraparesis, and dysphagia. A cerebral CT scan revealed acute posterior circulation territory ischemia. On the next day, the patient displayed pulselessness and cyanosis of lower extremities and elevated creatinine kinase (4,000 Units/L). An abdominal CT scan revealed embolism of the abdominal aorta, splenic and bilateral renal artery infarction, and multiple additional emboli in bilateral common and superficial femoral artery, left common iliac artery, and left popliteal artery. Emergency percutaneous embolectomy of aorta, bilateral iliac artery, right renal artery, and bilateral femoral artery was performed. Lysis catheters were placed in right and left popliteal arteries, and treatment with unfractionated heparin and urokinase was started and continued for one week. One day after initiation of local lysis, selective embolectomy of bilateral dorsal pedis and posterior tibial arteries was performed due to ongoing signs of lower extremity ischemia. Despite these efforts, the right lower leg had to be amputated one week later owing to irreversible ischemia. Ten days later, the patient developed right paraparesis due to acute media infarction confirmed by cranial CT scan. Three weeks prior to myocardial infarction, she was diagnosed with paralytic ileus, and ischemic segments of the small intestine were resected. Histological examination of embolic material removed from the right lower leg showed polygonal cells with eosinophilic cytoplasm and myxoid matrix, consistent with atrial myxoma. Transesophageal echocardiography (TEE) revealed an echogenic structure (1.0 × 0.5 × 0.5 cm) with a villous surface appearance in the left atrium attached to the fossa ovalis that was first diagnosed as atrial thrombus. At that time, the patient was deemed not suitable for open heart surgery due to a risk of intracerebral hemorrhage following media infarction, and she was scheduled for surgery 4 weeks later. Further retrospective anamnesis revealed that the patient was diagnosed with vasculitis 7 months earlier. Vasculitis was suspected owing to cachexia, night sweating, and elevated inflammatory markers including C-reactive protein (33.1–35 mg/L), leukocyte count (10.5–18.4 cells/nL), and erythrocyte sedimentation rate (69 mm/h). Furthermore, temporary weakness of the right arm and transient scotoma in the right eye suggested cerebral vasculitis, which was further supported by cranial magnetic resonance imaging revealing small lesions indicative of lacunar ischemia.

Following coronary angiography in our institution, the diagnosis of left atrial myxoma was confirmed by TEE (Figures 1(e) and 1(f)), and the patient was transferred to the department of cardiothoracic surgery. The myxoma was removed and atrial septum reconstruction was performed using a pericardial patch. During followup visits, the patient did not report any symptoms of embolism, and recurrence of left atrial myxoma was excluded by TEE two years after surgery.

## 3. Discussion

In summary, we report an extraordinary severe case of left atrial myxoma, highlighting the significance of systemic embolism as a sign of cardiac myxoma and emphasizing the requirement of early cardiac evaluation of these patients. In cases of suspected atrial myxoma, and particularly in patients presenting with recurrent arterial embolism, transesophageal echocardiography is mandatory. The detection of small atrial tumors that may not be apparent in transthoracic echocardiography is critical, as the tumor size does not correlate to the emboligenic potential. Diagnostic differentiation of myxoma from atrial thrombus is required. Multiple emboli may mimic systemic vasculitis, a typical but less common finding associated with cardiac myxoma. In order to prevent severe courses and to optimize clinical outcome, immediate surgical removal of myxoma is required as the probability of thromboembolic complications increases over time.
